# Number of endoscopic sessions to eradicate varices identifies high risk of rebleeding in cirrhotic patients

**DOI:** 10.1186/s12876-022-02283-0

**Published:** 2022-05-02

**Authors:** Huiwen Guo, Ming Zhang, Na Zhang, Xiaochun Yin, Yang Cheng, Lihong Gu, Xixuan Wang, Jiangqiang Xiao, Yi Wang, Xiaoping Zou, Yuzheng Zhuge, Feng Zhang

**Affiliations:** Department of Gastroenterology, Affiliated Drum Tower Hospital of Nanjing University Medical School, 321#, Zhongshan Road, Nanjing, 210008 Jiangsu China

**Keywords:** Number, Endoscopic therapy, Variceal rebleeding

## Abstract

**Background and aims:**

Risk stratification to identify patients with high risk of variceal rebleeding is particularly important in patients with decompensated cirrhosis. In clinical practice, eliminating gastroesphageal varices thoroughly after sequential endoscopic treatment reduces the rebleeding rate, however, no simple method has been build to predict high risk of variceal rebleeding. We conducted this study to explore the value of the number of endoscopic sessions required to eradicate gastroesphageal varices in identifying high risk of rebleeding.

**Patients and methods:**

Consecutive cirrhotic patients received sequential endoscopic therapy between January 2015 and March 2020 were enrolled. Endoscopic treatment was performed every 1–4 weeks until the eradication of varices. The primary endpoint was variceal rebleeding.

**Results:**

A total of 146 patients were included of which 60 patients received standard therapy and 86 patients underwent sequential endoscopic treatment alone. The cut-off value of the number of sequential endoscopic sessions is 3.5 times. Variceal rebleeding was significant higher in patients with endoscopic sessions > 3 times versus ≤ 3 times (61.5% vs. 17.5%, *p* < 0.001). Variceal rebleeding of patients with endoscopic sessions ≤ 3 times was significant lower than patients with > 3 times in group of standard therapy (19.6% vs. 88.9%, *p* < 0.001) and endoscopic therapy (15.9% vs. 47.1%, *p* = 0.028) respectively.

**Conclusion:**

The number of sequential endoscopic sessions required to eradicate the varices is related to the risk of variceal rebleeding in patients with cirrhosis. If three times of endoscopic treatment can not eradicate the varices, a more aggressive treatment such as TIPS should be seriously considered.

**Supplementary Information:**

The online version contains supplementary material available at 10.1186/s12876-022-02283-0.

## Introduction

Gastroesophageal varices (GOV) bleeding, one of the complications of portal hypertension, is a severe and life-threatening disease with a 6-week mortality up to 15–25% in patients with decompensated cirrhosis [1, 2]. A combination of non-selective beta-blockers (NSBBs) and endoscopic treatment is recommended as the first-line therapy for secondary prevention of variceal bleeding, while Transjugular Intrahepatic Portosystemic Shunt (TIPS) is recommended when the combination therapy fails [3]. Patients with variceal bleeding are often very dangerous, especially those who fail standard therapy, some of them do not even have a chance to switch to salvage TIPS. Therefore, risk stratification to identify patients with high risk of variceal rebleeding is particularly important in decompensated cirrhosis.

Portal pressure is closely related to the prognosis of patients with decompensated cirrhosis. Previous studies showed that hepatic venous pressure gradient (HVPG) reflected the severity of portal hypertension, and is still the "gold standard" for the diagnosis of portal hypertension and predicting prognosis [4, 5]. However, HVPG measurement is an invasive procedure with less compliance in both physicians and patients, and has certain requirements for the operator's technique and medical equipment with expensive cost as well [6]. Above all, adverse events such as transient arrhythmia and vagus nerve reaction may occur during the process of operation. These deficiencies largely limit the clinical application and promotion of HVPG.

Previous studies [7–9] have shown that the hemodynamic response of NSBBs can be used to identify the high risk of variceal bleeding, and in the secondary prevention of variceal bleeding, the rebleeding rate of HVPG responders (HVPG reduced to 12 mmHg or reduced to more than 20% baseline [7, 10, 11] is lower compared to nonresponders. However, some NSBBs hemodynamic responders still suffered GOV bleeding. It seemed that NSBBs response could not completely predict variceal bleeding, which indicated there might be other factors unrevealed. In addition, the biggest limitation of NSBBs response is still that it needs to be based on HVPG measurement. Therefore, whether NSBBs respond or not can not be used to predict the high risk of variceal bleeding completely.

As a non-invasive risk stratification tool, Child–Turcotte–Pugh (CTP) score is widely used in patients with variceal bleeding. The consensus of American Association for the Study of Liver Diseases(AASLD) 2016 [12] emphasized that, if there is no contraindication to TIPS, early TIPS can reduce the treatment failure rate and mortality of patients with CTP Grade C or CTP Grade B and active bleeding. However, the stratification tool is only suitable for patients with acute hemorrhage, and the role of CTP score in secondary prophylaxis is remained unknown. Therefore, it is necessary to explore a simple and effective method to predict high-risk rebleeding in cirrhotic patients with a history of variceal bleeding. In recent years, with the continuous development of endoscopic technology, endoscopic treatment of GOV bleeding has been widely used. Endoscopic treatment can effectively control acute varices bleeding, reduce rebleeding rate and prolong survival time [13]. The consensus of AASLD 2016 [12] emphasized that endoscopic treatment should be carried out sequentially until the varices are eradicated. However, even if the treatment goal is achieved, some patients still have recurrent varices and rebleeding in a short time, and the therapeutic effectiveness is poor.

In clinical practice, we observed that patients who needed more times of endoscopic sessions to achieve eradication of varices were more likely to bleed during the follow-up. Whether there is a correlation between the number of sequential endoscopic treatment and the cumulative rebleeding rate has not been revealed up to now. Therefore, we designed this study to explore the predictive value of the number of sequential endoscopic sessions required to eradicate varices in high-risk of variceal rebleeding.

## Patients and methods

### Patients

The patients in this study were screened from the prospective database of the Department of Gastroenterology in Nanjing Drum Tower Hospital between January 2015 and March 2020. The inclusion criteria was as follows: (1) Aged more than 18 years old; (2) Cirrhosis diagnosed based on clinical symptoms combined with laboratory or image examinations and presence of GOV type 1 (GOV1)/GOV type 2 (GOV2) and esophageal varices (EV) determined by esophagogastroduodenoscopy (EGD) before secondary prevention; (3) Treated with endoscopic therapy for secondary prevention; (4) Written informed consent was obtained. The exclusion criteria of the study was as follows: (1) Sequential endoscopic therapy was not standardized; (2) Previous TIPS was performed; (3) Concomitant malignant tumors; (4) Failure of sequential endoscopic therapy: rebleeding before eradication of varicose veins; (5) Severe heart, lung, liver, kidney dysfunction; (6) Women who were pregnant or breastfeeding; (7) Missing follow-up data.

### Definitions

The standard endoscopic therapy was defined as recommended in the 2016 AASLD consensus [12], that endoscopic variceal ligation (EVL) or endoscopic injection sclerosis(EIS) or endoscopic tissue glue injection or combination of them was performed every 1–4 weeks until the eradication of varices. The number of endoscopic sessions was the times of endoscopic treatment needed to eradicate varices (no further ligation possible) [3]. First EGD review was performed 3–6 months after eradication and every 6–12 months thereafter [12]. Failure of standard endoscopic therapy was defined as that variceal rebleeding occurs in patients who had not achieve complete varices eradication during sequential treatment. Recurrence of GOV was defined as the observation of new varices after eradication had been achieved [14].

### Study protocol

**Fig. 1:**
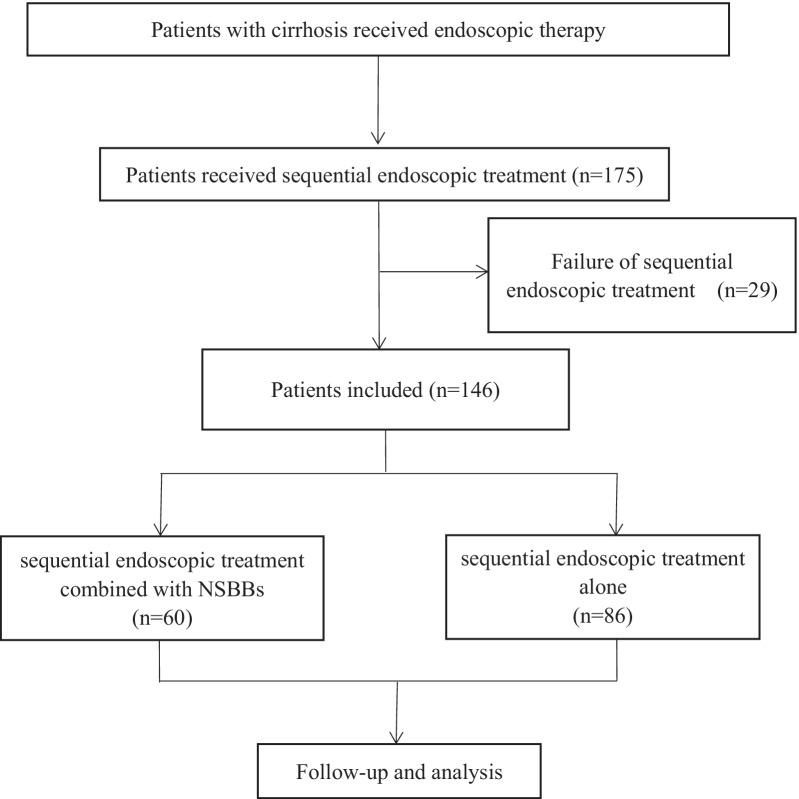
Flow chart of patients involved

Patients with GOV bleeding were screened for this single-centre retrospective cohort study. In accordance with the inclusion and exclusion criteria, a total of 175 patients received sequential endoscopic treatment and 29 patients switched to TIPS or liver transplantation due to endoscopic treatment failure. Eventually, 146 patients were included in the analysis of which 60 patients received sequential endoscopic treatment combined with NSBBs and 86 patients underwent sequential endoscopic treatment alone (Fig. 1). The 60 patients took NSBBs and adjusted the dosage according to the guidelines [3]. Endoscopic treatment was performed every 1–4 weeks until the eradication of varices (no further ligation possible). Postoperative conditions such as rebleeding or death were monitored and recorded during the follow-up. The study protocol, which conformed to the the principles of the 1975 Declaration of Helsinki, was approved by the Ethics Committee of our center. All patients provided written informed consent before endoscopic treatment.

### Endoscopic therapy

All patients were fasted for 6–8 h before operation. After general anesthesia, they were performed routine EGD under electrocardiograph (ECG) monitoring. Patients with EV were treated with EVL or EIS or in combination and with gastric varices were treated with EIS or tissue glue injection or in combination according to the willingness of patients, the experience of doctors and the conditions of blood vessels comprehensively. All of the varices were high risk, with the shape of F2–F3 and the presence of red color signs. The patient was treated under intravenous anesthesia. After the operation, fasting and antacids were given and vital signs were monitored. Then liquid diet was gradually given and transitioned to soft food. Re-check the EGD every 1–4 weeks, and perform endoscopic treatment again if necessary until the varices are eradicated. The ligator is a six-shot ligator (MET; COOK Company), the main component of the hardener and tissue glue is lauromacrogol injection (lauryl alcohol polyoxyethylene) from TIANYU PHARMACEUTICAL Company and n-butyl α-cyanoacrylate adhesive, from COMPONT Company.

### Follow-up

Patients’ follow-up was performed mainly by EGD to record the condition of varices and details of clinical events of patients. The last follow-up was ended on December 2020. The primary endpoint was GOV bleeding, defined as recommended in the Baveno VI consensus [3], and the secondary endpoint was variceal recurrence and liver transplant-free survival.

### Statistical analysis

SPSS version 25.0 (SPSS Inc., Chicago, Illinois, USA) software was used for all data statistics. Quantitative data are presented as mean ± standard deviation (SD) if conforming to normal distribution and presented as median (range) if not conforming to normal distribution. Classification variables were expressed as counts and percentages. Independent sample *t*-tests and Chi-square tests were used to compare the differences of patients between different groups. A receiver operating characteristic (ROC) curve analysis was performed to calculate the sensitivity, specificity, positive and negative predictive values. The number of sequential endoscopic sessions with the best specificity and sensitivity (Youden’s Index) was chosen to optimize the predictive ability of GOV bleeding. The cumulative probability of the patients who exhibited GOV bleeding was evaluated via Kaplan–Meier curves and histogram. The univariate and multivariate Cox proportional hazards models were used to detect independent predictors of GOV bleeding. Statistical significance was established at *p* < 0.05.

## Results

### Patients’ baseline characteristics

The baseline characteristics are shown in Table 1. Ninety (61.6%) of the patients were male. The mean age of the patients was 54 years old (range, 22–88 years). Median number of sequential endoscopic sessions was 2. Median follow-up was 21.25 months. During the whole follow-up, 4 patients died, including one due to uncontrolled bleeding and three due to hepatocellular carcinoma. Three patients underwent liver transplantation. Rebleeding occurred in 37 patients (25.3%) and all the bleeding were originated from GOV. After rebleeding, 27 patients still received endoscopic treatment, 5 patients received conservative treatment, 4 patients switched to TIPS, and 1 patient died because of uncontrolled bleeding.

**Table 1:** Patient demographics, liver disease characteristics, and clinical presentation (median and ranges)

Variables	Overall population (n = 146)
Age (year)	54 (22–88)
Sex (female/male)	56/90
Etiology of liver cirrhosis (viral/others)	62/84
CTP score	7 (5–11)
CTP classification (A/B/C)	68/73/5
PT (s)	13.65 (9.3–24.6)
TB (µmol/L)	15.85 (4.4–138.7)
Ascites (No/Mild/Moderate/Large)	27/46/46/27
Number of sequential endoscopic sessions	2 (1–7)
Median follow-up time(m)	21.25 (95% CI, 20.114–24.467)

CTP, Child-Turcotte-Pugh; PT, prothrombin time; TB, total bilirubin

Endoscopic treatment included endoscopic tissue glue injection, EIS and EVL. 82 patients had EV alone and 64 patients had EV combined with gastric varices (28 with GOV1 and 36 with GOV2). 60 patients were treated with sequential endoscopic therapy combined with NSBBs, that is, standard treatment. Among them, 27 patients were administrated propranolol and 33 patients took carvedilol.

### Predicting factors associated with variceal rebleeding

We took age, gender, etiology of cirrhosis, CTP score, prothrombin time (PT), total bilirubin (TB), creatinine (Cr), ascites and number of sequential endoscopic sessions into univariate analysis and found that only the number of sequential endoscopic sessions was significantly associated with the cumulative variceal rebleeding. Furthermore, we included the number of endoscopic sessions and clinically meaningful TB and CTP scores into multivariate analysis, and finally indicated that the number of sequential endoscopic sessions was an independent predictor of variceal rebleeding (OR 1.408; 95% CI, 1.122–1.767, *p* = 0.003) (Table 2). ROC curve (Additional file 1: Fig. S1) and Youden’s Index was performed to optimize the predictive ability of GOV bleeding. The biggest Youden index was 0.34 and AUROC was 0.694 (0.587–0.801). The cut-off value of the number of sequential endoscopic treatment is 3.5 times, and the sensitivity and specificity were 43.2% and 90.8% respectively.

**Table 2:** Univariate and multivariate analysis for predicting factors associated with variceal rebleeding after sequential endoscopic treatment

Variable	Univariate analysis		Multivariate analysis	
	HR (95% CI)	*P* value	HR (95% CI)	*P* value
Age (year)	0.991 (0.965–1.016)	0.477		
Sex (male)	0.791 (0.397–1.577)	0.505		
Etiology of cirrhosis	1.182 (0.615–2.270)	0.616		
CTP score	0.999 (0.789–1.264)	0.992	0.957 (0.719–1.275)	0.765
PT (s)	0.946 (0.808–1.107)	0.486		
TB (µmol/L)	1.007 (0.990–1.024)	0.425	1.005 (0.985–1.024)	0.642
Cr (µmol/L)	1.006 (0.990–1.022)	0.470		
*Ascites*				
No	Reference			
Mild	1.765 (0.601–5.182)	0.301		
Moderate	1.349 (0.512–3.559)	0.545		
Large	1.108 (0.401–3.058)	0.843		
Number of sequential endoscopic sessions	1.417 (1.133–1.773)	0.002	1.408 (1.122–1.767)	0.003

CTP, Child-Turcotte-Pugh; PT, prothrombin time; TB, total bilirubin; Cr, creatinine

### Over three times of endoscopic sessions needed to eradicate varices predicts high-risk of variceal rebleeding

**Fig. 2:**
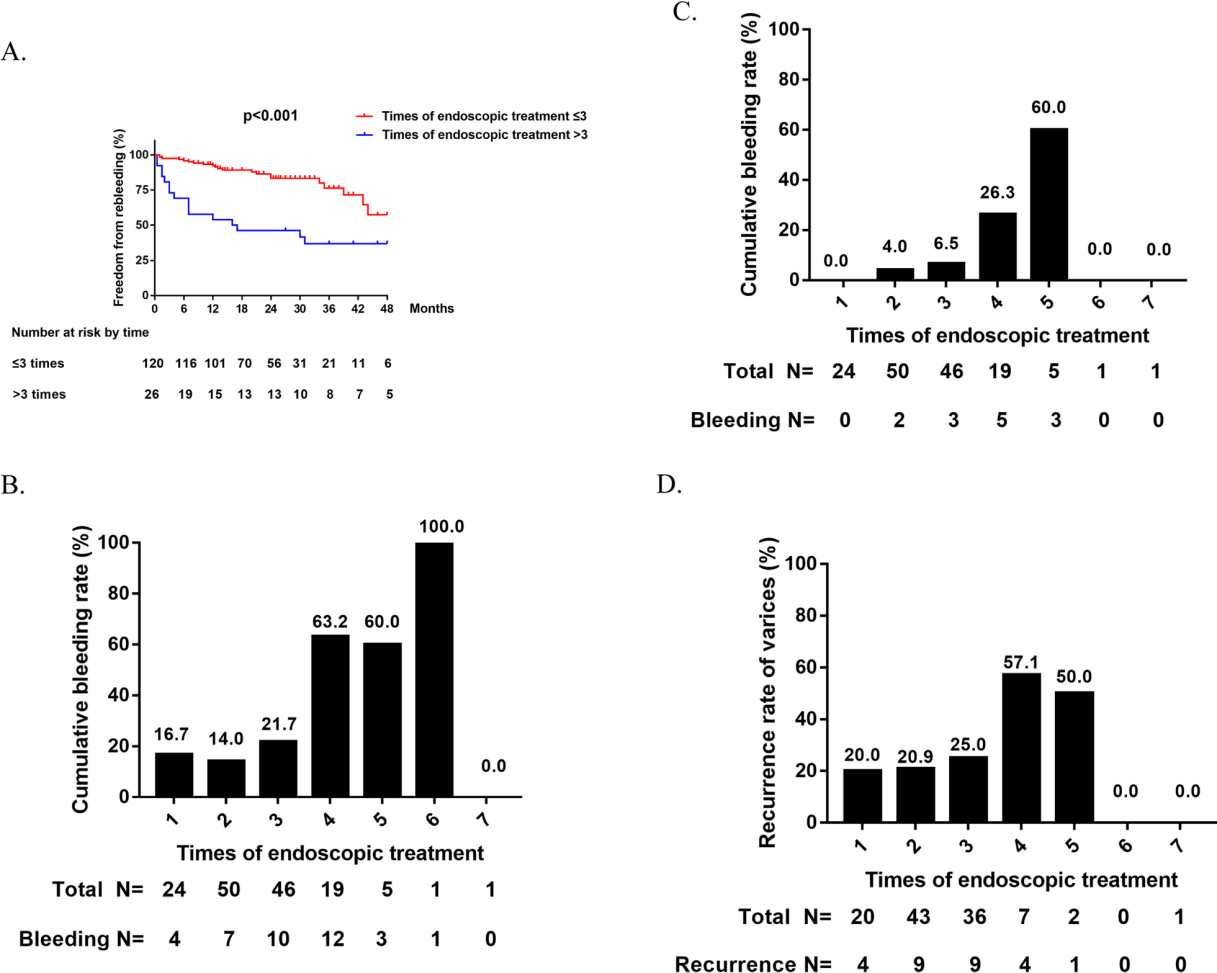
**A** Comparison of variceal rebleeding rate between patients with number of endoscopic sessions ≤ 3 times and > 3 times. **B** Comparison of variceal rebleeding rate between patients with different number of endoscopic sessions during the whole follow-up period. **C** Comparison of short-term variceal rebleeding rate between patients with different number of endoscopic sessions. **D** Comparison of recurrence rates of varices between patients with different number of endoscopic sessions

All patients were divided into two groups of ≤ 3 times and > 3 times according to the number of sequential endoscopic sessions based on the best cut-off value. Among them, 120 (82.2%) patients needed ≤ 3 times of endoscopic sessions to eradicate varices and the remaining 26 (17.8%) patients needed over 3 times. There was no significant difference in baseline characteristics between the two groups, such as age, gender, CTP score, ascites, etc. (Additional file 2: Table S1). During the follow-up, 21 (17.5%) patients in the group of ≤ 3 times experienced variceal rebleeding compared to 16 (61.5%) patients in the group of > 3 times. There was a significant difference in the cumulative overall variceal rebleeding rate between the two groups (*p* < 0.001, Fig. 2A). The median rebleeding time of the two groups was 13.0 months and 5.5 months respectively and was different significantly (Z =  − 2.148, *p* = 0.016). According to the number of sequential endoscopic sessions, the cumulative rebleeding rate of varices in patients with 1–5 times of treatment was 16.7%, 14%, 21.7%, 63.2% and 60% respectively (Fig. 2B) and the 6-month rebleeding rate was 0%, 4%, 6.5%, 26.3% and 60% respectively (Fig. 2C). The recurrence of varices during the dynamic review of EGD were 20.0%, 20.9%, 25%, 57.1% and 50% for patients who underwent 1–5 times endoscopic treatment (Fig. 2D). In total, the outcomes of patients with ≤ 3 times of endoscopic sessions needed to eradicate varices were quite different from those with > 3 times of sequential endoscopic sessions.

### Number of endoscopic sessions is independent on NSBBs in predicting variceal rebleeding

**Fig. 3:**
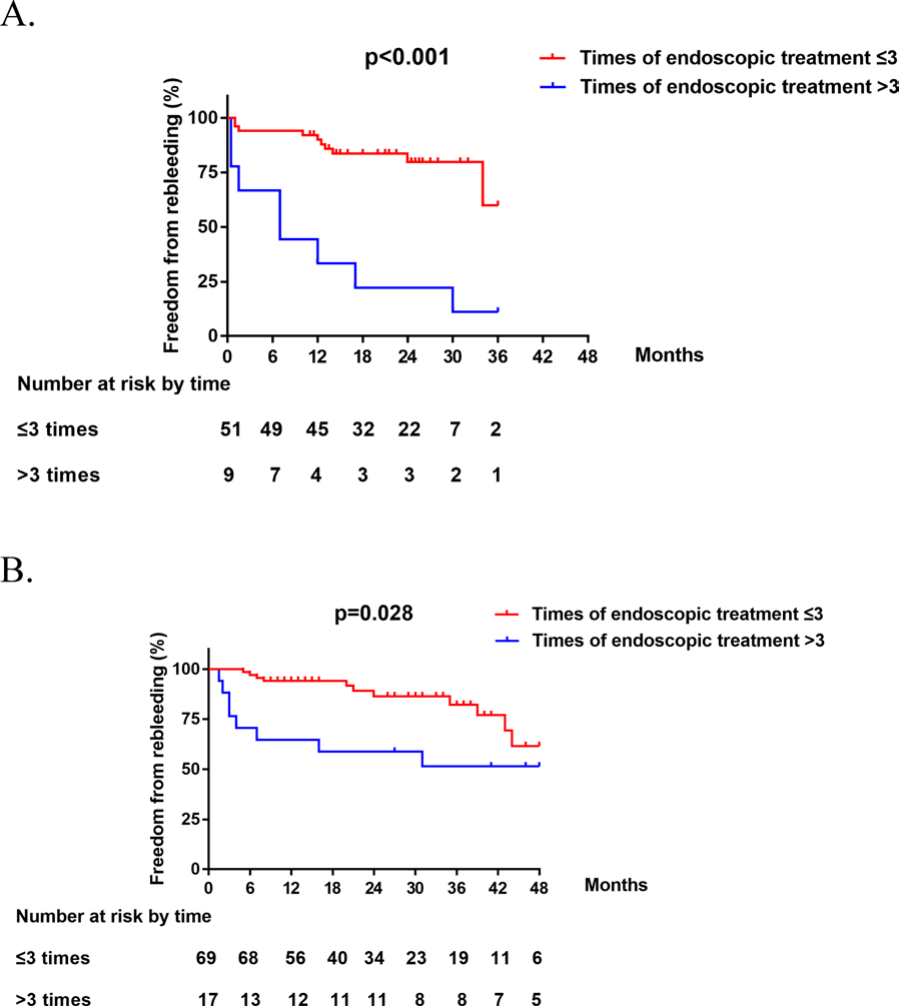
**A** Comparison of variceal rebleeding rate between patients with number of endoscopic sessions ≤ 3 times and > 3 times in group of standard therapy. **B** Comparison of variceal rebleeding rate between patients with number of endoscopic sessions ≤ 3 times and > 3 times in group of sequential endoscopic treatment alone

All of the enrolled patients were divided into groups of sequential endoscopic therapy combined with NSBBs (n = 60) and sequential endoscopic therapy alone (n = 86). The variceal rebleeding of patients with endoscopic sessions ≤ 3 times was significant lower than patients with more than 3 times in group of standard therapy (19.6% vs. 88.9%, *p* < 0.001, Fig. 3A) and endoscopic therapy (15.9% vs. 47.1%, *p* = 0.028, Fig. 3B) respectively. The results were similar to those of the whole cohort analysis.

## Discussion

The Baveno VI guidelines proposed and emphasized the treatment strategies of portal hypertention should be based on risk stratification [3]. Risk stratification methods included non-invasive and invasive tools. The former was commonly used involving CTP score and model for end-stage liver disease (MELD) score, etc., while the latter, such as HVPG. Some studies have shown that early TIPS can effectively reduce the variceal rebleeding rate and improve the survival rate in patients with Child–Pugh Grade C (less than 14 scores) and Child–Pugh Grade B complicated with active bleeding [12, 15]. However, the significance of CTP score in secondary prevention of variceal bleeding has not been verified. On the other hand, although the role of HVPG in the diagnosis of portal hypertension and guiding stratified therapy has been emphasized [12], as an invasive procedure, HVPG has serious compliance, availability and cost problems, and some patients have shunt when measuring HVPG, which severely limit its application in routine clinical practice [16].

There are some studies to evaluate portal hypertension through biochemical examination, doppler ultrasound, shear wave elastography (SWE), magnetic resonance imaging (MRI) and other noninvasive techniques [3, 12, 17]. The laboratory biomarker can not diagnose or exclude portal hypertension at least now, and other noninvasive detection methods such as liver and spleen stiffness, magnetic resonance elastography (MRE) combined with plasma platelet count have shown satisfactory evidence in the diagnosis of portal hypertension, but these studies have not yet covered the area of risk stratification that guides the treatment of portal hypertension [18–20]. Therefore, to establish a convenient, simple and efficient clinical stratified tool for secondary prevention, the number of endoscopic sessions needed to eradicate varices is worth of further study.

As far as we know, this study is the first time to reveal that the number of endoscopic sessions can identify the cirrhotic patients with high risk of variceal rebleeding in sequential endoscopic treatment for secondary prevention of GOV bleeding. In patients enrolled, the variceal rebleeding of patients with sequential endoscopic sessions > 3 times was significantly higher than that of patients with sequential endoscopic sessions ≤ 3 times and number of endoscopic sessions is independent on NSBBs in predicting variceal rebleeding. Therefore, sequential endoscopic sessions for three times as a risk stratification tool has potential value.

In our study, the overall variceal rebleeding was 25.3% (37/146) which was similar to the bleeding rate in the previous studies that ranged from 7.8 to 29% [21–28]. The recurrence rate of varices in patients without rebleeding was 29.4% (32/109), which was lower than previous study [29]. This may be due to the development of endoscopic technology in recent years and the fact that a significant proportion of patients in our study were treated with sequential endoscopic therapy in combination with NSBBs. Of the total 146 patients, 60 patients were treated with NSBBs combined with sequential endoscopy, and 86 were treated with sequential endoscopy alone. The results were consistent with the whole population, which confirmed that number of endoscopic sessions was independent on the use of NSBBs in predicting variceal rebleeding. Therefore, it is suitable for the patients who underwent sequential endoscopic treatment alone or combined with NSBBs.

Although endoscopic therapy combined with NSBBs is recommended as the first-line treatment in the guidelines for secondary prevention of GOV bleeding in cirrhotic patients, it is difficult to achieve the standard treatment for all patients in the real world for all sorts of reasons, such as NSBBs intolerance or contraindications, poor compliance of patients and so on. There are still some problems in the real world needed further discussion in the future. First of all, some patients without contraindications of NSBBs don’t use NSBBs in a standardized way; Secondly, sequential endoscopic treatment is not standardized that some patients do not receive sequential treatment or the time interval can not meet the requirements of the guidelines.

A number of studies have shown that standardized sequential endoscopic therapy can effectively control variceal bleeding, and reduce or eradicate varices to prevent rebleeding [15, 21, 30]. In our study, the median number of endoscopic treatment to eradicate varices was 2 times, which was similar to the previous literature [22, 23, 31]. Therefore, our results are comparable to other studies. We conducted COX univariate and multivariate analysis and found that only the number of endoscopic treatment is an independent risk factor for variceal rebleeding rate. Therefore, according to the number of endoscopic treatment, patients at high risk of variceal rebleeding can be identified.

We confirmed the importance of the number of endoscopic sessions in cirrhotic patients. However, several limitations still can not be ignored. Firstly, our study was retrospective and conducted at a single centre. However, all data were taken from prospective databases. Secondly, endoscopic treatment was not uniform, which may affect the final results. Furthermore, only some patients were administrated NSBBs, but the results of the subgroup analysis were consistent with the overall analysis that the variceal rebleeding rate was higher in patients with more than 3 times endoscopic sessions. More rigorous multicenter prospective studies may be required to validate our results in the future.

In conclusion, the number of sequential endoscopic sessions required to eradicate the varices is related to the risk of variceal rebleeding in patients with cirrhosis. When three times of endoscopic treatment can not eradicate the varices, a more aggressive treatment such as TIPS should be sought as soon as possible.

## Supplementary Information

**Additional file 1**.  Receiver operating characteristic (ROC) curve of number of endoscopic sessions in predicting high risk of rebleeding.

**Additional file 2**. Patient demographics, liver disease characteristics, and clinical presentation between groups (median and ranges).

## Data Availability

All data generated or analysed during this study are included in this published article and its Additional files 1, 2.
