# Supplementary material for: Number of endoscopic sessions to eradicate varices identifies high risk of rebleeding in cirrhotic patients
Source: BMC Gastroenterol. 2022 May 2;22:213. doi: 10.1186/s12876-022-02283-0 (PMC9063156; doi:10.1186/s12876-022-02283-0)
Supplement: Supplementary file 1 — Additional file 1. Receiver operating characteristic (ROC) curve of number of endoscopic sessions in predicting high risk of rebleeding. [file 12876_2022_2283_MOESM1_ESM.docx]

Supplemententary Fig 1. Receiver operating characteristic (ROC) curve of number of endoscopic sessions in predicting variceal rebleeding.
