# Supplementary material for: Number of endoscopic sessions to eradicate varices identifies high risk of rebleeding in cirrhotic patients
Source: BMC Gastroenterol. 2022 May 2;22:213. doi: 10.1186/s12876-022-02283-0 (PMC9063156; doi:10.1186/s12876-022-02283-0)
Supplement: Supplementary file 2 — Additional file 2. Patient demographics, liver disease characteristics, and clinical presentation between groups (median and ranges). [file 12876_2022_2283_MOESM2_ESM.docx]

**Supplementary Table 1 Patient demographics, liver disease characteristics, and clinical presentation between groups (median and ranges)**

| Variables | Times of endoscopic treatment≤3 (n=120) | Times of endoscopic treatment>3 (n=26) | P value |
| --- | --- | --- | --- |
| Age (year) | 53.5 (22-88) | 54.5 (27-81) | 0.638 |
| Sex (female/male) | 49/71 | 7/19 | 0.266 |
| Etiology of liver cirrhosis (viral/others) | 49/71 | 13/13 | 0.512 |
| CTP score | 7 (5-11) | 7 (5-11) | 0.408 |
| CTP classification (A/B/C) | 57/60/3 | 11/13/2 | 0.405 |
| PT (s) | 13.65 (9.3-24.6) | 13.8 (10.6-19.4) | 0.482 |
| TB (umol/L) | 15.95 (4.4-73.2) | 14.25 (10.3-138.7) | 0.335 |
| Ascites (No/Mild/Moderate/Large) | 25/36/38/21 | 2/10/8/6 | 0.416 |
| Median follow-up time(m) | 22 (1-48) | 16.5 (0.5-48) | 0.239 |

CTP, Child-Turcotte-Pugh; PT, prothrombin time; TB, total bilirubin.
